# Biological Roles and Clinical Applications of Exosomes in Breast Cancer: A Brief Review

**DOI:** 10.3390/ijms25094620

**Published:** 2024-04-24

**Authors:** Han Wang, Ruo Wang, Kunwei Shen, Renhong Huang, Zheng Wang

**Affiliations:** Department of General Surgery, Comprehensive Breast Health Center, Ruijin Hospital, Shanghai Jiao Tong University School of Medicine, Shanghai 200025, China; wanghan8844@163.com (H.W.); wangruo@sjtu.edu.cn (R.W.); kwshen@medmail.com.cn (K.S.)

**Keywords:** breast cancer, exosomes, extracellular vesicles, tumor microenvironment

## Abstract

Breast cancer (BC) is a global health risk for women and has a high prevalence rate. The drug resistance, recurrence, and metastasis of BC affect patient prognosis, thus posing a challenge to scientists. Exosomes are extracellular vesicles (EVs) that originate from various cells; they have a double-layered lipid membrane structure and contain rich biological information. They mediate intercellular communication and have pivotal roles in tumor development, progression, and metastasis and drug resistance. Exosomes are important cell communication mediators in the tumor microenvironment (TME). Exosomes are utilized as diagnostic and prognostic biomarkers for estimating the treatment efficacy of BC and have the potential to function as tools to enable the targeted delivery of antitumor drugs. This review introduces recent progress in research on how exosomes influence tumor development and the TME. We also present the research progress on the application of exosomes as prognostic and diagnostic biomarkers and drug delivery tools.

## 1. Introduction

Breast cancer (BC), one of the most commonly detected malignant tumors in women, is a major cause of cancer-associated deaths globally [1,2,3,4]. BC exhibits high heterogeneity and is classified into several subtypes according to genetic and clinical characteristics, including luminal A, luminal B, triple-negative breast cancer (TNBC), and HER2 enrichment [5,6,7,8,9]. Despite advances in the early detection of BC and the availability of effective treatment methods, including radiotherapy, mastectomy, and chemotherapy, BC has been reported to show high metastasis, invasion, and recurrence rates and high drug resistance [10,11,12,13,14,15]. Poor prognosis due to distant organ metastasis and treatment failure are the major hindrances to successfully treating patients with advanced-stage BC [16,17,18]. Hence, the key molecular mechanisms underlying the poor prognosis of BC patients need to be clarified.

Exosomes are cell-synthesized lipid bilayer vesicles [19], with a 30–150 nm particle size [20]. Exosome biogenesis and secretion into the extracellular space involve initiation, endocytosis, multivesicular body formation, and final release [21]. Exosomes are commonly detected in several body fluids, including urine, blood, saliva, amniotic fluids, and breast milk, under normal physiological and pathological conditions [22,23]. A critical function of exosomes is the transport of various functional components, such as lipids, proteins, mRNAs, microRNAs (miRNAs), noncoding RNAs (ncRNAs), and DNA fragments, to the extracellular space [24]. These exosomal components induce phenotypic changes in receptor cells and mediate intercellular communication locally and globally in the body [25,26,27]. Thus, by affecting adjacent or distant cells, exosomes participate in regulating immune function, antigen presentation, cell proliferation, angiogenesis, and tumor cell invasion and migration [28,29,30]. Extracellular vesicles (EVs) are critically involved in the immune suppression and chemotherapeutic resistance of BC cells [31,32]. Because of the rich biological information available on this topic, exosomes, which are involved in liquid biopsies, have the potential for their utilization as biomarkers for the early diagnosis, efficacy detection, and prognosis prediction of BC patients [33,34,35]. Additionally, because of the unique structure of exosomes, drugs delivered through exosomes have better biocompatibility than other delivery methods, thus making them a potential treatment option [36].

**Figure 1:**
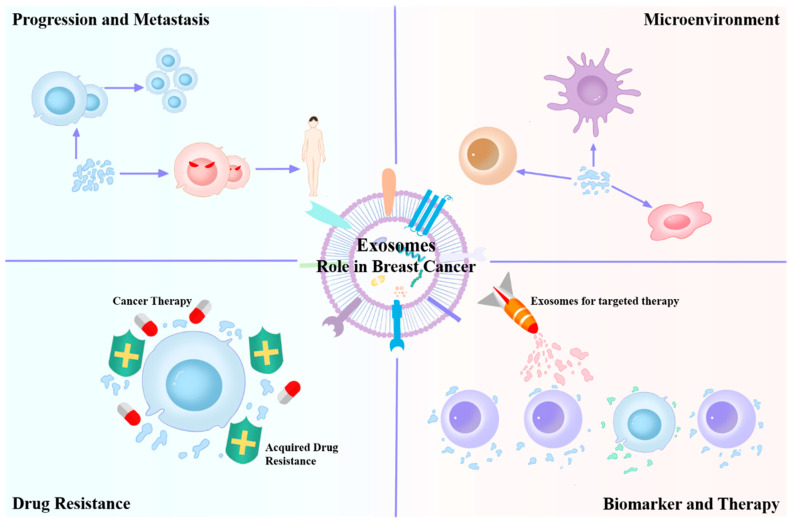
Multiple roles of exosomes in various aspects of breast cancer.

This paper presents a brief review on the research on the impact of exosomes (Figure 1) on tumor development and the tumor microenvironment (TME). We also review recent findings on the application of exosomes as prognostic and diagnostic biomarkers and drug delivery tools. The literature review method can be found in the Supplementary Materials, Table S1.

## 2. Cancer-Derived Exosomes Can Advance Tumor Progression and Metastasis

Exosomes exhibit pleiotropic roles in BC metastasis and progression, including angiogenesis, invasion, and immune regulation. According to Deng et al., BC cell-derived exosomes can enhance the in vivo metastasis of BC, activate focal adhesion kinase/Src-dependent proteins, and increase proinflammatory cytokine and MMP9 production [37]. Kong et al. detected miR-130a-3p downregulation in BC tissues and circulating exosomes; they also observed an association between the decreased exosome-derived miR-130a-3p level and lymph node (LN) metastasis and advanced TNM stage [38]. According to in vitro and in vivo experiments, exosomal miR-222 contributes to BC cell tumorigenicity and metastasis, possibly through the downregulation of the tumor suppressor genes *PDZ* and *PDLIM2* as well as the subsequent activation of NF-kB [39]. BC cell-derived exosomes are enriched in miR-1910-3p, which can be transferred to BC and breast epithelial cells through MTMR3 regulation and NF-κB signaling pathway activation, thereby facilitating BC cell growth and metastasis [40]. Pan et al. reported that exosomal miR-145 derived from MDA-MB-231 cells targets IRS1 and inhibits HUVEC angiogenesis by controlling the IRS1/Raf/ERK pathways and IRS1/PI3K/Akt/mTOR [41]. TNBC cell-derived exosomal miR-939 enhanced the cross-endothelial migration of tumor cells and targeted vascular endothelial cadherin directly in endothelial cells. Exosomal miR-939 from BC cells participates in extracellular tumor-promoting features and shows an association with the poor prognosis of TNBC patients [42]. A close interaction of the TME with cancer cells is necessary for metastatic potential development. Previous research has shown that cancer-derived exosomes deliver proteins, lipids, and ncRNAs to surrounding cells or cells present in distant metastatic niches through premetastatic TME regulation. As confirmed by Wang et al., exosomal miR-4443 overexpression strongly enhanced liver metastasis in a mouse BC metastasis model. Exosomal miR-4443 delivery can disrupt the natural barrier, accompanied by damage to TIMP2, leading to MMP-2 upregulation in the liver metastatic lesion and the primary tumor, resulting in transplantation into a new microenvironment [43]. In a mouse lung metastasis model, exosomal miR-183-5p inhibited the transfer of PPP1CA to macrophages, thereby promoting IL-2 and TNF-α (proinflammatory cytokines) secretion [44]. The exosomal AnxA2 level in malignant cells was significantly greater than that in normal cells as well as premetastatic BC cells. Additionally, an in vivo analysis revealed that depletion of the EV AnxA2 reduced the number of metastatic sites in the lungs and brain, which confirmed the critical role of AnxA2 in forming a TME that favors metastasis [45].

**Figure 2:**
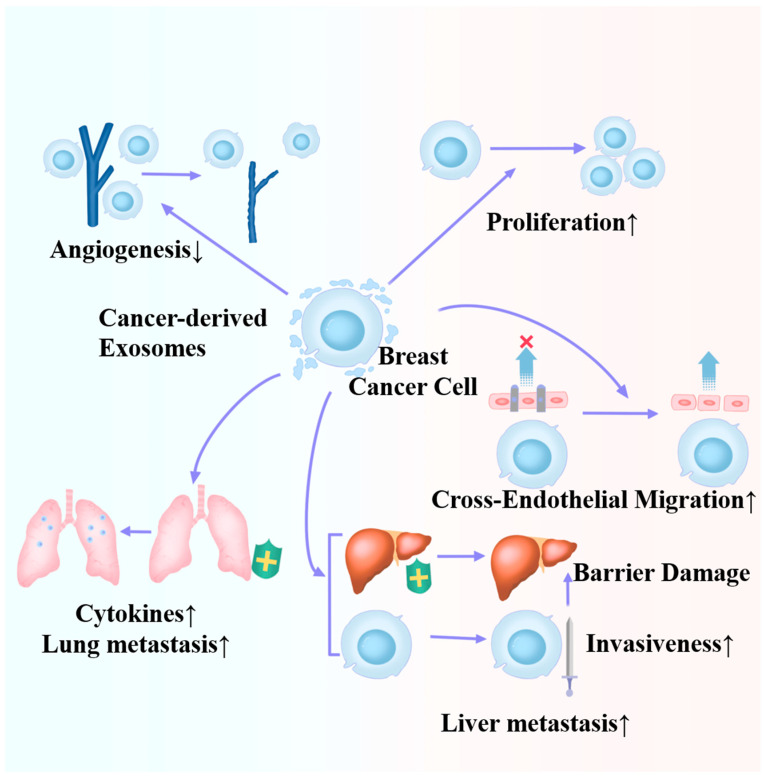
Roles of cancer-derived exosomes in the metastasis and progression of breast cancer.

Table 1 and Figure 2 depict an overview of exosome progression and metastasis in BC. In fact, although a considerable extent of research focuses on how exosomes promote breast cancer progression or metastasis, not all exosomes are harmful, and miR-145 is a typical example. Therefore, subsequent research directions should focus on the study of exosomes, which is beneficial for treating diseases to develop better tools for BC treatment. We also noticed that the role of exosomes seems to not be limited to the original tumor itself but also affects other organs. This effect is currently suggested to be detrimental to patients in the current study, but could it be beneficial? Therefore, this reminds us of the necessity of systematic research in in vivo studies. It is worth noting that although some studies have suggested proteomic differences in exosomes, a large number of studies have focused on differences in exosome content rather than exosome quantity [46,47]. This may suggest potential future research possibilities.

## 3. Roles of Cancer-Derived Exosomes in the BC Microenvironment

BC-derived exosomes contain rich biological information and have substantial involvement in the cellular communication and biological regulation of tumor cell interactions with the surrounding microenvironment [48,49]. Following induction by BC-derived exosomes, cells residing in the BC microenvironment may influence the biological characteristics of BC through various ways.

Fibroblasts exhibit high abundance in the typical TME [50]. They are spindle-shaped and secrete various extracellular matrix (ECM) proteins that maintain the connective tissue framework and assist in wound healing [51,52]. In the TME, fibroblasts are significantly involved in tumor cell interactions with surrounding cells [53]. Cancer-associated fibroblasts (CAFs) are a crucial cell subtype involved in the coevolution of BC cells and their microenvironment through cellular compartment modification and stromal–epithelial interaction-mediated cancer cell regulations [54]. Tumor cell-derived exosomes can transform normal fibroblasts (NFs) to CAFs. According to previous studies, exosomal miR-9, miR-125b, etc., from BC cells, can induce fibroblasts to produce CAF-like characteristics [55,56]. miR-105 exerts a protumor effect by mediating stromal cell metabolism reprogramming. miR-105 also mediates the metabolic reprogramming of CAFs, thereby providing energy to neighboring cancer cells through the enhancement of glucose and glutamine metabolism under nutrient-rich conditions. Under nutrient-deficient conditions, metabolic waste, such as lactic acid, is converted into energy to support tumor growth [57]. Ahn et al. noted that luminal A cancer cell-derived exosomes delivered miR-130b-3p to CAFs and promoted BC progression by downregulating SPIN90 [58]. Wang et al. revealed that, in BC lung metastasis, tumor cells transfer Cav-1 to the metastatic organ microenvironment through exosomes, which promotes tenascin-C secretion in lung fibroblasts and induces ECM deposition. It also inhibited the PTEN/CCL2/VEGF-A signaling pathway in lung macrophages and promoted angiogenesis and M2-type macrophage polarization, thereby mediating intercellular communication and regulating the premetastatic niche of lung metastasis [59]. BC cells show a high expression of survivin, which is delivered to fibroblasts through exosomes to upregulate SOD-1 expression in CAFs; this provides a feedback to tumor cells, resulting in the enhancement of their metastasis and proliferation [60].

Tumor-associated macrophages (TAMs) are an important group of immune response-associated cells in the BC microenvironment that have recently received considerable attention [61,62]. Macrophages can transform from a tumor-suppressing state to a tumor-promoting state through M1 to M2 polarization [63,64,65]. BC cell-derived exosomes can influence the microenvironment by affecting the polarization process. According to in vitro studies, BC cell-derived exosomes can transfer GP130 to macrophages and alter their polarization through STAT3 pathway activation [66]. miR-138-5p, from BC cells, is delivered to TAMs through exosomes and can decrease KDM6B expression, suppress M1 polarization, activate M2 polarization, and promote lung metastasis in BC patients [67]. Lin28B facilitates the lung metastasis of BC cells. It shows an upregulated expression in the exosomes of TNBC patients and facilitates neutrophil recruitment and M2 polarization, which is critical for PD-L1 upregulation in lungs and for immunosuppression due to a cytokine imbalance [68]. The tumor exosomal circRNA cSERPINE2 is significantly elevated in BC and controls the MALT1-NF-κB-IL-6 axis in TAMs; it also increases IL-6 secretion that facilitates BC cell proliferation and invasion [69]. Endoplasmic reticulum (ER) stress stimulates exosomal Circ_0001142 release from BC cells, which regulates tumor progression by triggering M2 macrophage polarization through the Circ_0001142/miR-361-3p/PIK3CB pathway [70]. ER stress also induces an increase in miR-27a-3p, which is then transferred to macrophages through exosomes and induces the MAGI2/PTEN/PI3K axis-mediated PD-L1 upregulation in TAMs [71]. BC cell-derived exosomes transfer miR-63 to TAMs by binding to the CD375 receptor. miR-375 also directly targets TNS3 and PXN, which increases tumor migration and infiltration by TAMs [72]. In vivo models of BC cell-derived exosomes can also transmit CCL5 to affect immune infiltration in the TME and promote lung metastasis in patients with BC [73].

Cancer-related adipocytes (CAAs) have also recently received a considerable amount of attention from researchers based on the analysis of patient serum-derived exosomes [62]. Zhao et al. noted that BC cell-derived exosomes activate CAA by delivering miR-1304-3p, accelerating adipocyte differentiation and lipid accumulation, targeting the antiadipogenic gene *GATA2* to exert a pro-carcinogenic effect, and potentially enabling the prediction of the prognosis of African American patients [74]. BC cell-derived exosomes deliver miR-155 to target UBQLN1 in adipocytes, inhibit white adipose tissue browning, and promote fat reduction related to cancer cachexia [75]. Exosomal miR-122 suppresses the uptake of glucose by premetastatic ecotone cells through the attenuation of pyruvate kinase (a glycolytic enzyme) expression, thus promoting disease progression [76]. In ER-positive BC, exosomal miR-19a is significantly upregulated and is delivered to osteoclasts through IBSP to induce the generation of osteoclasts, thus forming a microenvironment conducive to tumor cell colonization [77]. BC cell-derived exosomes also stimulate bone marrow cell differentiation into MDSCs by downregulating CXCR4 expression and activating the STAT3 signaling pathway, which is closely associated with the drug resistance and poor prognosis of BC patients [78].

**Figure 3:**
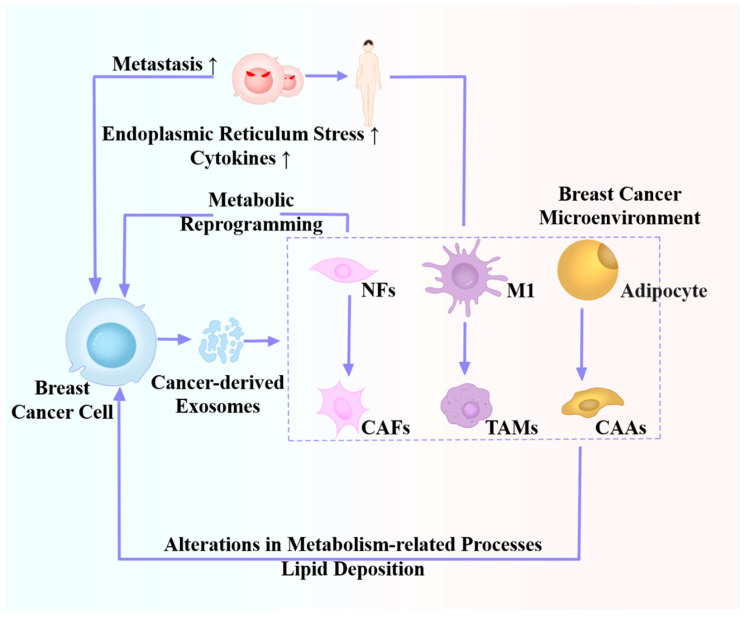
Roles of cancer-derived exosomes in the microenvironment of breast cancer cells.

Table 2 and Figure 3 show the relevant mechanisms underlying the effect of BC-driven exosomes on the TME. Current research on the roles of cancer-derived exosomes in the BC microenvironment mainly focuses on CAF, TAM, CAA, and other cells. However, the mechanism by which cancer-derived exosomes produce CAA cells is still unclear. In addition, there are a large number of different tumor-related cells in the TME, and the relationship between these cells and roles of cancer-derived exosomes deserves to be explored. Based on current research progress, future research should focus on the following: (1) are there any beneficial effects of BC-driven exosomes on the TME? and (2) can a specific target be developed to block exosomes for therapeutic purposes?

## 4. Roles of Microenvironment-Derived Exosomes in BC

### 4.1. Effects of Fibroblast-Derived Exosomes

Exosomal miRNAs are essential mediators of the interaction of tumor cells with CAFs. CAF-derived exosomes containing miR-181d-5p increase the aggressive behavior of BC cells by targeting the CDX2/HOXA5 axis [79]. miR-1b and miR-3-7p are delivered to BC cells from CAFs through exosomes, which enhance tumor cell metastasis and invasion by downregulating TCEAL1 and GLIS232 [80]. The upregulation of miR-18b in CAF-derived exosomes stimulates NF-kB activation through specific binding to TCEAL7, promotes nuclear Snail ectopic activation to induce EMT, and induces cellular metastasis and invasion [81]. CAF-derived exosomes target LATS2 through the miR-92/PD-L1 axis, which enhances BC cell proliferation and migration and impairs tumor-infiltrating immune cell functions [82]. Chen et al. validated miR-500a-5p’s role in CAF-derived exosomes and confirmed that its effect on promoting proliferation was achieved by targeting USP28 [83].

The proteins enriched in exosomes also participate in intercellular communication. Fibroblasts with a low p85α expression can change the TME and promote BC metastasis by the exosome-mediated parasecretion of Wnt10b [84]. ADAM10-rich exosomes derived from CAFs can facilitate cellular motility and activate the RhoA and Notch signaling pathways in BC cells, thus affecting tumor progression [85]. Oxidized ATM promotes the phosphorylation of BNIP3, thus inducing the accumulation of the autophagy-related protein GPR64 in hypoxia CAF-derived exosomes, promoting IL-8 and MMP9 upregulation in BC cells and enhancing cancer cell invasiveness [86].

### 4.2. Effects of Immune Cell-Derived Exosomes

Macrophages, a highly heterogeneous group of immune cells, exhibit strong plasticity in the TME. As shown previously, TAMs promote BC metastasis, and high-density TAMs are an adverse prognostic factor for BC. Exosomal miR-503-3p generated from macrophages upregulated DACT2, inhibited glycolysis through the Wnt/β-catenin signaling pathway, and promoted oxidative phosphorylation in the mitochondria of BC cells [87]. In vivo experiments revealed that TAM-derived exosomal miR-223-3p can be delivered to 4T1 cells and facilitate lung metastasis in BC patients by targeting Cbx5 [88]. Yu et al. investigated how TAM-derived exosomes influence tumor recurrence and metastasis after chemotherapy. Following the coculturing of THP-1-derived macrophages and apoptotic BC cells, TAM-secreted exosomes enhanced BC cell invasion, migration, and proliferation by activating *STAT3* and its target genes *CyclinD1*, *MMP2*, and *MMP9* [89]. Evidence from other studies also suggests that TAMs facilitate tumor progression [64,90]. TAM-derived exosomes delivered Circ_0020256, which targets miR-432-5p and E2F3 to increase the activity of cholangiocarcinoma cells and promote cholangiocarcinoma development [91]. Prostate cancer (PCa) cells can uptake miR-95 delivered by TAM-derived exosomes, and the binding of miR-95 with its downstream target gene *JunB* promotes PCa cell invasion, proliferation, and epithelial-mesenchymal transition (EMT); moreover, the high miR-95 expression level is associated with worse clinicopathological features [92].

### 4.3. Effects of Adipocyte-Derived Exosomes

Compared to that of immune cells and fibroblasts, the role of adipocytes in tumor progression has been less studied. Recent studies have increasingly appreciated the role of adipocytes in the TME. Wang et al. noted that adipocyte-derived exosomes can stimulate BC cell proliferation and migration through the Hippo signaling pathway [93]. Studies on BC patients with type 2 diabetes revealed that CAA-derived exosomes promote the expression of genes associated with cancer stem cell and EMT traits. TSP5 was upregulated in exosomes and related to EMT. COMP co-expression with BRD2 or BRD3 indicated poor patient prognosis [94]. Gernapudi et al. confirmed that preadipocyte-derived exosomes can affect the differentiation, migration, and stemness of BC cells through the miR-140/SOX2/SOX9 axis [95]. Related studies on other cancers confirmed the role of CAA-derived exosomes in tumor progression. Adipocyte-derived exosomes are specifically enriched in proteins involved in fatty acid oxidation (FAO) and can induce the metabolic reprogramming of tumor cells, which is beneficial for FAO to stimulate melanoma cell invasion and migration [96]. Adipocytes increase MMP9 activity in tumor tissues by producing exosomes with a high MMP3 level, thereby promoting lung cancer cell invasion in vivo and in vitro [97].

**Figure 4:**
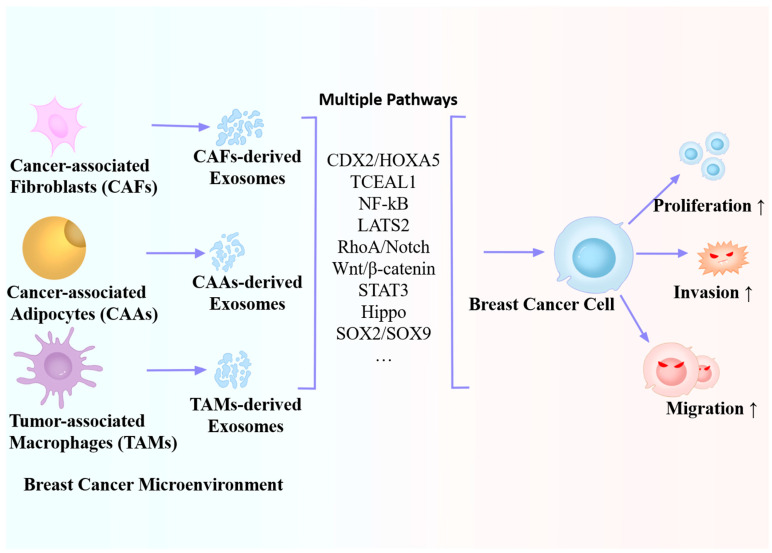
Roles of microenvironment-derived exosomes in the proliferation, invasion, and migration of breast cancer.

Table 3 and Figure 4 show the effects of microenvironment-driven exosomes on BC cells. To summarize, in the BC microenvironment, microenvironment-driven exosomes or BC cell-driven exosomes jointly form a closed loop between BC and the microenvironment; however, many studies have analyzed only one aspect. Therefore, future studies should investigate its overall regulatory mechanism.

## 5. Roles of Exosomes in BC Drug Resistance

### 5.1. Exosomes in Chemoresistance

Chemotherapy is a critical component of comprehensive BC treatment. Efflux and the inactivation of drugs are the primary mechanisms of drug resistance caused by exosomes in BC. According to previous studies, the chemotherapeutic drug doxorubicin is excreted through vesicles secreted by cells [98]. P-gp is transported by exosomes from docetaxel-resistant BC cells to docetaxel-sensitive cells, thereby inducing acquired docetaxel resistance [99]. Adriamycin-resistant MCF-7 cell-derived exosomes have a rich content of the proteins P-gp and UCH-L1. The treatment of sensitive MCF-57444 cells with LDN-1 (a UCH-L1-specific inhibitor) can prevent EV internalization-induced resistance in adriamycin-resistant MCF-7 cells [100]. Paclitaxel (PTX) treatment induces MDA-MB-231 cells to secrete survivin-rich exosomes and strongly promotes the survival of PTX-treated SK-BR-3 cells and fibroblasts [101]. Exosomes generated from MDA-MB-231 cells resistant to cisplatin show high miR-423-5p expression. Cisplatin-resistant cells are transferred to recipient cells through the promotion of proliferation, metastasis, and antiapoptotic signaling [102]. According to Wang et al., high exosomal lncRNA-H19 levels induced a resistance to doxorubicin in BC cells. Furthermore, lncRNA-H19 inhibition remarkably reduced a resistance to doxorubicin [103]. Exosome miR-1246 promotes a resistance to docetaxel, gemcitabine, and epirubicin through Cyclin-G2 inhibition in BC cells [104].

### 5.2. Exosomes in Hormone Resistance

Approximately 70% of patients with BC show tumors containing a high level of estrogen receptor-α (ERα); therefore, a hormonal therapy that targets ERα is an effective approach. Semina et al. reported that TAM-resistant MCF-7 cell-derived exosomes caused horizontal hormone resistance in MCF-7 cells with an estrogen dependency. A coculture of sensitive MCF-7 cells for 14 days with exosomes derived from drug-resistant cells caused the sensitive cells to show antiestrogen drug resistance [105]. An exosomal transfer of the lncRNA UCA1 induced ER-MCF-7 cells to exhibit a resistance to TAM through the mTOR signaling pathway [106]. A downregulation of miR-222 increases ERα and p27 expressions at the mRNA and protein levels and restores the sensitivity of cells to TAM; furthermore, exosomal miR-222 may enhance the resistance to TAM by upregulating ERα and p27 in ER-BC cells [107]. Trastuzumab-resistant BC cells secrete exosomes containing SNHG14, which inhibits apoptosis through the Bcl-2-associated pathway and induces trastuzumab resistance [108]. Exosomes containing the AGAP2-AS1 lncRNA also enhance BC cell resistance to trastuzumab [109].

### 5.3. Resistance of Exosomes to HER2-Targeted Therapy

Targeting HER2 is an important part of the comprehensive treatment of BC; however, targeted resistance is also an important reason for HER2-positive BC progression. HER2-targeted drug resistance is linked with the elevated levels of PD-L1 and TGF-β1. Martinez observed that exosomes transfer these molecules to characterize their cell of origin in drug-sensitive cells. Exosome-derived TGF-β1 levels were related with the response of patients with HER2-related BC to HER2-targeted therapy, thus suggesting the utilization of this cytokine as a treatment response biomarker [110]. The miR-567 expression was downregulated during trastuzumab resistance, and exosomal miR-567 reversed the resistance to trastuzumab by autophagy inhibition; however, miR-567 knockdown induced trastuzumab resistance [111].

Unlike research studies, the clinical treatment approach commonly involves the combination of multiple treatments (e.g., a chemotherapy combination or sequential targeted therapy). Therefore, exosome-related resistance mechanisms are worth exploring in the combination of multiple treatments. The second possible research direction is that, currently, many research investigations focus on cancer-driven exosomes, and a more holistic view is the related role of microenvironment-driven exosomes in drug resistance. In addition, in advanced BC treatments, including fulvestrant, a resistance to CDK4/6 inhibitors cannot be ignored. There may be an exosome-related mechanism, which deserves further studies.

## 6. Potential Clinical Applications of Exosomes in Treating BC

### 6.1. Exosomes as Potential Biomarkers of BC

Exosomes contain rich biological information, and exosome testing is an effective method for liquid biopsies with the advantages of noninvasiveness and dynamic monitoring. An increasing number of tumor diagnostic or prognostic markers are being developed. An increase in the concentration of the Hsp70 protein is a characteristic of tumor exosomes. Del-1, fibronectin, 20S proteasome, and spliced survivin are also present in the blood exosomes of patients with BC [112]. Compared to healthy women, BC patients show a lower CD82 level on the circulating exocrine body surface. The low expression of the aforementioned four transmembrane proteins in exosomes is associated with the metastatic characteristics of tumors [113]. Studies of serum-derived exosomes have shown a higher expression of molecules such as CD24, FAK, EGFR, and GPC-1 in BC patients than in healthy individuals; thus, these molecules have the potential to function as diagnostic and prognostic biomarkers [114,115,116]. Several miRNAs in circulating exosomes have emerged as potential biomarkers to enable the early detection of BC and to differentiate BC subtypes. An Au nanoprobe can differentiate BC patients from healthy people by detecting miRNA-1246 [117]. The analysis of the miRNA expression profiles of exosomes revealed the following findings: (1) the different subtypes of BC populations had specific miRNA expression profiles, (2) HER2-positive BC patients and TNBC patients had specific miRNA expression patterns in plasma exosomes, and (3) the miRNA expression was associated with clinicopathological parameters and pCR [118]. Studies have also shown that long RNAs in exosomes can predict clinical outcomes. Among BC patients receiving neoadjuvant therapy, those who achieved pCR showed a differential expression of 2573 exosomal long RNAs as compared to patients with residual lesions. Further analyses revealed that MSMO1 expression levels in exosomes were linked with the clinical outcomes of BC patients [119].

Research on exosome biomarkers is progressing rapidly. However, current traditional exosome extraction and isolation technologies do not seem to meet the clinical requirements for the highly sensitive extraction and isolation of exosomes required for liquid biopsies. Therefore, future research may need to focus on industrialized exosome extraction and isolation methods.

### 6.2. Exosomes in BC Treatment

Although a variety of treatments are already used for the treatment of cancer, more treatments still need to be developed [120,121,122,123]. Exosomes are independent units with a complete membrane structure. Surface membrane proteins can enhance the endocytosis of receptor cells. Exosomes can be designed to target the delivery of anticancer drugs or other customized molecules without causing toxicity to target cells. PTX-loaded exosomes can be obtained after sufficient incubation with MSCs; these exosomes effectively inhibit cancer cell growth and prevent tumor angiogenesis in bones [124]. Metastatic BC 4T1 cell-derived exosomes can effectively deliver doxorubicin to mice lungs and inhibit lung metastasis [125]. Tumor-derived exosomes can use transcytosis to cross the blood–brain barrier, thus showing that exosomes could be used as drug delivery vehicles to target the brain for treating BC brain metastases [126]. Park et al. designed exosomes that deliver three miRNAs, namely, miR-19a-3p, miR-19b-3p, and miR-1226-3p, which inhibit BC cell migration by targeting AQP5 [127]. α-Lactalbumin-engineered BC exosomes loaded with human neutrophil elastase and hiltonol enhanced immunogenic cell death processes and promoted antitumor immune properties in TNBC [128]. Li et al. modified the exosome surface with an anti-HER2 antibody and an anti-CD3 antibody to target HER2-positive BC cells and activate T-cell immunity [129]. Activated tumor-associated effector T cell-derived exosomes carry membrane-bound PD-1, which enhances T-cell cytotoxicity to TNBC cells by occupying PD-L1 and weakening the subsequent dysfunction of T cells [130]. Engineered macrophage-secreted exosomes were developed, modified, and loaded with DOX using peptides targeting the mesenchymal-epithelial transition factor. These engineered exosomes significantly prolong the DOX circulation time, which specifically targets tumors, promotes apoptosis, and exhibits low hepatotoxicity [131]. Macrophage-tumor chimeric exosomes can be enriched in LNs through a direct interaction with exosomes, effectively leading to tumor regression and extending the survival of a mouse tumor recurrence model when combined with anti-PD-1 therapy [132].

Although exosomes have great promise as a therapeutic measure, there are still many issues that limit their future applications: (1) What kind of cells should be selected as the donor of exosomes? (2) How should the targeting of exosomes be modified? (3) How should drug loading efficiency be improved? (4) How should the engineered production of exosomes be realized?

## 7. Conclusions and Outlook

Exosomes contribute to tumor progression, TME remodeling, tumor metastasis promotion, and drug resistance and have been studied in depth in BC. Recent investigations have partially elucidated the mechanism through which exosomes participate in intercellular communication and TME remodeling. Active components in exosomes, including miRNAs, proteins, and lipids, are involved in tumorigenesis, which is achieved through the promotion of EMT, angiogenesis, vascular permeability, and metastatic prebiotic regulation. However, because of the complex composition of the TME, which contains multiple cell types and secreted factors, EVs secreted by different cells can synergistically promote tumor occurrence and metastasis. Therefore, it is difficult to determine which cell types of exosomes or cytokines play a major role. Exosomal miRNAs are considered critical mediators of intercellular communication. However, only the differences in the expression levels of these genes in tissue samples have been investigated, and there is a lack of in-depth mechanistic research.

Traditional screening methods for BC include ultrasounds, mammography, MRIs, etc. Compared to traditional methods, a liquid biopsy serves as a noninvasive and rapid detection method. Tumor-derived exosomes widely occur in various body fluids. Exploring exosome biomarkers is helpful for an early BC diagnosis. In addition, EV biomarkers can dynamically reflect disease conditions and predict patient prognosis. However, there is no clear method to determine the molecular subtype from exosomes. Tumor-derived exosomes can also serve as therapeutic targets, and blocking the delivery or release of exosomes or their key components is a promising therapeutic approach. The construction of engineered exosomes used for targeted drug delivery provides a new direction for future personalized therapy.

## Figures and Tables

**Table 1:** Cancer-derived exosomes in the metastasis and progression of breast cancer.

Biological Roles	Intermediates	Major Mechanisms	References
BC metastasis	/	FAK/Src-dependent proteins	[37]
BC progression	miR-130a-3p	RAB5B	[38]
BC invasiveness and metastasis	miR-222	PDZ/PDLIM2/NF-kB	[39]
BC progression and metastasis	miR-1910-3p	MTMR3/NF-kB	[40]
HUVEC angiogenesis (BC inhibition)	miR-145	IRS1/Raf/ERK IRS1/PI3K/Akt/mTOR	[41]
BC cross-endothelial migration	miR-939	Vascular endothelial cadherin	[42]
BC liver metastasis	miR-4443	Barrier damage TIMP2/MMP-2	[43]
BC lung metastasis	miR-183-5p	PPP1CA-IL2/TNFα	[44]

**Table 2:** Cancer-derived exosomes in the different tumor microenvironment cell subtype and breast cancer cells.

TME Cell Subtype	Biological Roles	Intermediates	Major Mechanisms	References
Cancer-associated fibroblasts				
	NF to CAF	miR-9	/	[55]
	NF to CAF	miR-125b	/	[56]
	BC tumor growth	miR-105	Metabolism reprogramming	[57]
	BC progression	miR-130b-3p	SPIN90	[58]
	BC lung metastasis	Cav-1	Extracellular matrix deposition	[59]
	BC metastasis and proliferation	Survivin	SOD-1	[60]
Tumor-associated macrophages				
	M1 to M2	GP130	STAT3 pathway	[66]
	M1 to M2	miR-138-5p	KDM6B	[67]
	BC lung metastasis	Lin28B	PD-L1/Cytokine imbalance	[68]
	M1 to M2	Circ_0001142	Endoplasmic reticulum	[70]
	BC immune escape	miR-27a-3p	PD-L1	[71]
	BC migration and infiltration	CD375/miR-63	TNS3PXN	[72]
	BC lung metastasis	CCL5	Immune infiltration	[73]
Cancer-related adipocytes				
	BC progression	miR-1304-3p	GATA2	[74]
	BC inhibition	miR-155	White adipose tissue browning	[75]
	BC progression	miR-122	Pyruvate kinase	[76]

**Table 3:** Tumor microenvironment-derived exosomes in breast cancer cells.

Tumor Microenvironment-Derived Exosomes	Biological Roles	Intermediates	Major Mechanisms	References
Fibroblast-derived exosomes				
	BC invasiveness	miR-181d-5p	CDX2/HOXA5 axis	[79]
	BC metastasis and invasion	miR-1b/miR-3-7p	TCEAL1 GLIS232	[80]
	BC metastasis and invasion	miR-18b	TCEAL7/NF-kB/Snail/EMT	[81]
	BC proliferation and migration	miR-92	PD-L1/LATS2	[82]
	BC tumor-infiltrating immune cell function			
	BC proliferation	miR-500a-5p	USP28	[83]
	BC metastasis	Wnt10b parasecretion		[84]
	BC progression	ADAM10	RhoA/Notch axis	[85]
	BC invasiveness	GPR64	IL-8/MMP9	[86]
Immune cell-derived exosomes				
	BC progression	miR-503-3p	DACT2/Wnt/β-Catenin	[87]
	BC lung metastasis	miR-223-3p	Cbx5	[88]
	BC recurrence and metastasis after chemotherapy	/	STAT3	[89]
	Cancer progression	Circ_0020256	miR-432-5p/E2F3	[91]
	Cancer progression	miR-95	JunB/EMT	[92]
Adipocyte-derived exosomes				
	BC cell proliferation and migration	/	Hippo	[93]
	BC cancer stem cell	TSP5 COMP/BRD2 or BRD3	EMT	[94]
	BC differentiation, migration, and stemness	miR-140	SOX2/SOX9	[95]
	Cancer invasiveness and migration	Proteins involved in fatty acid oxidation	Metabolic reprogramming	[96]
	Cancer invasiveness	MMP3	MMP9	[97]

## Data Availability

Not applicable.

## Supplementary Materials

The supporting information can be downloaded at: https://www.mdpi.com/article/10.3390/ijms25094620/s1.

## Abbreviations

BC	Breast cancer
TNBC	Triple-negative breast cancer
ncRNAs	Noncoding RNAs
miRNAs	MicroRNAs
TME	Tumor microenvironment
CAFs	Cancer-associated fibroblasts
NFs	Normal fibroblasts
TAMs	Tumor-associated macrophages
CAAs	Cancer-associated adipocytes
FAO	Fatty acid oxidation
Erα	Estrogen receptor-α
