# Supplementary material for: Biological Roles and Clinical Applications of Exosomes in Breast Cancer: A Brief Review
Source: Int J Mol Sci. 2024 Apr 24;25(9):4620. doi: 10.3390/ijms25094620 (PMC11083446; doi:10.3390/ijms25094620)
Supplement: Supplementary file 1 [file ijms-25-04620-s001.zip › ijms-2940419-supplementary.pdf]

Supplementary Materials

Biological roles and clinical applications of exosomes in breast cancer: A brief review

Han Wang[1, †], Ruo Wang[1, †], Kunwei Shen[1], Renhong Huang[1, *] and Zheng Wang[1, *]

1. Department of General Surgery, Comprehensive Breast Health Center, Ruijin Hospital, Shanghai Jiao Tong University School of Medicine, Shanghai 200025, China
† These authors contributed equally.
Email: hrhyisheng@163.com; wilsonwangzheng@163.com

Searching Methods

The following Medical Subject Headings (MeSH) terms were used to search the PubMed, Web of Science, Medline, etc. databases: “Breast Cancer” and “Exosome”. Articles with those terms, published in English were retrieved. Google Scholar was also reviewed to retrieve additional articles. The relevant literature was searched using the following keywords: “Progression”, “Metastasis”, “Microenvironment”, “Drug Resistance”, “Biomarker” and “Therapy”, respectively. The research selection process was divided into the following three stages: title review, abstract review and full-text review. Original articles and review articles appropriate to the topic of this review were included in the full-text review phase. The search strategy is detailly illustrated in **Table S1**.

Table S1. The search strategy summary.

Items	Specification
Date of search	July 15, 2023
Databases and other sources searched	PubMed, Web of Science, Medline, Google Scholar, etc.
Search terms used	Breast Cancer, Exosome, Progression, Metastasis, Microenvironment, Drug Resistance, Biomarker and Therapy
Inclusion and exclusion criteria	Inclusion criteria: The search was limited to articles published in English. The research selection process was divided into the following three stages: title review, abstract review, and full-text review. Original articles and review articles appropriate to the topic of this review were included in the full-text review phase.
Selection process	H Wang and R Wang searched the articles, scored them independently, and eliminated articles with scores in the bottom 25%. Subsequently, RH Huang and Z Wang independently scored the articles again, and only articles with comprehensive scores higher than the norm were selected for literature review. KW Shen supervised the entire process. The scoring criteria are mainly based on: the scientific significance of the article, the innovative value of the article and the data of the article.
